# Impact of service improvement measures in a Victorian paediatric eating disorders program

**DOI:** 10.1186/2050-2974-2-S1-O14

**Published:** 2014-11-24

**Authors:** Suba Rudolph, Kellie Draffin, Shea Edsall, Saadia Oularbi

**Affiliations:** 1 Department of Paediatrics, Austin Health, Melbourne, Australia; 2 Department of Nutrition and Dietetics, Austin Health, Melbourne, Australia

## 

Three interventions were implemented to improve the paediatric eating disorders service at the Austin Hospital. The primary changes consisted of (i) introduction of a multidisciplinary initial assessment clinic, (ii) increased utilisation of Family-Based Treatment (FBT), and (iii) institution of aggressive inpatient feeding. A retrospective audit from 2011, 2012 and 2013 was conducted extracting medical records data. Service referrals doubled (28 in 2011 to 58 in 2013), associated with greater acuity at initial presentation (50% vs 65% at <90% expected body weight). The vast majority of admissions for medical stabilisation occurred within 2 weeks of presentation, reflecting this greater acuity (78% vs 92%). Readmission rates halved (33% to 17%) with a dramatic 75% reduction in patients requiring >3 admissions (33% to 8%). A 59% uptake of FBT by 2013 led to substantial improvements in weight restoration, from 46% to 71% of patients achieving >90% expected body weight within 6 months. Increased utilisation of FBT is expected to further improve outcomes. FBT patients were more likely to achieve complete weight restoration (odds ratio 1.7). This escalating number and severity of service referrals demands additional resources and funding if the complex psychiatric and medical needs of our patients are to be adequately addressed.

This abstract was presented in the **Service Initiatives: Child and Adolescent Refeeding and FBT** stream of the 2014 ANZAED Conference.

